# Clinical Effects and Feasibility of Virtual Reality Table Tennis in People With Parkinson’s Disease in a Supervised Outpatient Setting: A Case Series

**DOI:** 10.7759/cureus.109454

**Published:** 2026-05-22

**Authors:** Shinsuke Fujioka, Koichi Nagaki, Junichi Takami, Kazuki Kimura, Yoshiki Yamaguchi, Kosuke Kitano, Hitoshi Kikuchi, Yoshio Tsuboi

**Affiliations:** 1 Neurology, Fukuoka University Faculty of Medicine, Fukuoka, JPN; 2 Neurology, Murakami Karindoh Hospital, Fukuoka, JPN; 3 Rehabilitation, Murakami Karindoh Hospital, Fukuoka, JPN

**Keywords:** parkinson's disease, quality of life, rehabilitation, table tennis, virtual reality

## Abstract

In Parkinson’s disease, conventional rehabilitation has long been a cornerstone of symptom management; however, in recent years, an increasing number of studies have reported the effectiveness of virtual reality (VR)-based rehabilitation. Although a variety of VR modalities have been explored, there have been no prior reports specifically investigating rehabilitation using table tennis-based VR software. We report a case series evaluating VR table tennis as rehabilitation in 4 patients with Parkinson’s disease (Hoehn-Yahr stages II-III). Participants completed 16 sessions of VR training. Motor and non-motor symptoms, quality of life (QOL), and usability were assessed using the MDS-UPDRS, PDQ-39, and System Usability Scale. Improvements exceeding the minimal clinically important difference were observed in motor function and QOL. All participants completed the program without encountering any adverse events. Our findings suggest that VR table tennis is a safe and feasible rehabilitation method for patients with Parkinson’s disease.

## Introduction

Continuous physical activity is important for people with Parkinson's disease (PwPD) as it assists them in maintaining physical and mental functions and quality of life (QOL) [1-3]. For PwPD, conventional rehabilitation has long been a cornerstone of symptom management; however, in recent years, an increasing number of studies have reported the effectiveness of virtual reality (VR) based rehabilitation [4]. Although a variety of VR modalities have been explored, there have been no prior reports specifically investigating rehabilitation using table tennis-based VR software. This study aimed to evaluate the clinical effects and feasibility of rehabilitation using a VR table tennis program for PwPD.

## Case presentation

We consecutively recruited participants until 4 PwPD (Hoehn-Yahr (HY) stages ≥II and <IV during the ’On’ stage) had been enrolled (P01, P02, P03, P04). Participation criteria included a clinical diagnosis of Parkinson’s disease made by a movement disorder specialist (SF), age ≥20 years. Participants were prohibited from using any other VR devices during the study period. They were functionally independent and had reported no falls in the past 12 months, and none had undergone deep-brain stimulation. Patients with severe visual impairment, vestibular disorders, epilepsy, marked susceptibility to motion sickness, severe freezing of gait, or severe dyskinesia were excluded because of safety and feasibility concerns related to VR-based rehabilitation. Demographic characteristics of the participants are shown in Table 1.

**Table 1:** Patients’ demographic characteristics DD=disease duration; HY= Hoehn & Yahr; MoCA=Montreal Cognitive Assessment

ID	Gender	Age (years)	Handedness	DD (years)	HY stage	MoCA	Medications
P01	F	78	Right	6	3	23	Rasagiline, Levodopa/carbidopa, Opicapone
P02	M	68	Right	7	2	28	Levodopa/carbidopa, Istradefylline, Safinamide
P03	M	74	Right	12	3	23	Levodopa/carbidopa, Zonisamide, Rotigotine, Symmetrel
P04	F	75	Right	5	3.5	25	Levodopa/carbidopa, Ropinirole patch, Istradefylline, Safinamide

This protocol combined VR table tennis rehabilitation with occupational therapy and speech therapy for the target patients. The VR model used was "Oculus Quest 2" along with "Eleven Table Tennis" software provided by Meta Platforms, Inc. Participants used a practice mode in which they rallied with a game opponent on a virtual table tennis table, and a match mode in which they played against an opponent. First, the patient performed a 5-minute preparatory exercise session [neck stretch, upper limb stretches, lower limb stretches, lateral step, and swinging (fore-back-switch)], followed by 15 minutes in practice mode. Then, after a 5-minute break, the participants played in match mode for 15 minutes. During training in match mode, breaks were taken at the patient's request or at the discretion of the assisting medical personnel. Patients at risk of falling due to balance problems could play the game in a seated position. Forty-minutes of occupational therapy and 40-minutes of speech therapy were given after the VR training. If the patient could not continue for more than 25 minutes due to fatigue or being off medication during a session, we excluded the session from statistical analyses.

The difficulty level of the VR task was adjusted across sessions based on the participant’s performance to maintain task engagement and safety, following the built-in adaptive settings of the VR system. All participants played against standardized virtual opponents provided by the VR application, ensuring consistent task conditions across sessions. Participants performed the VR task using their dominant hand, and hand dominance was recorded for all individuals. Exercise intensity and caloric expenditure were not objectively monitored during VR sessions, which represents a limitation of the present study.

All four participants were tested with the following outcome measures before and after the 16 exercise sessions during an “on medication” state. Baseline measures were age, sex, height, weight, education, duration of illness, modified HY stage, ratio of non-fallers/fallers in the past six months, Montreal Cognitive Assessment (MoCA) score [5], Movement Disorders Society Unified Parkinson's Disease Rating Scale (MDS-UPDRS) [6], and levodopa equivalent daily dose. The PDQ-39 was used to assess patients' QOL [7]. The primary outcome measure was the MDS-UPDRS part III score, which indicated motor symptoms. Secondary outcomes included MDS-UPDRS part I, part II, and part IV, which indicated the ability of non-motor symptoms, ADL, and motor complications, respectively. PDQ-39 summary index was also used for the secondary outcome. All participants were assessed during the on-medication phase at 2 intervals: 1-week before exercise, and after 8-weeks of exercise. A further endpoint was 8-week class attendance. After the study period, we evaluated the usability of, and satisfaction with, VR table tennis using the System Usability Scale (SUS), which was created as a satisfaction evaluation index. For the assessment of feasibility, we checked the rate of attendance and adverse events.

To confirm the validity of the assessment scales used for physical therapy, we used minimal clinically important difference (MCID), based on the consensus-based standards for the selection of health measurement instruments, an international standard for scale development. Because the current study included a small number of participants, we followed the above-mentioned recommendation and used the MCID to evaluate each outcome. The results of MDS-UPDRS part I, part II, part III, part IV, and PDQ-39 were evaluated according to the MCID to assess whether the exercise therapy had a meaningful effect [8-11]. Outcome assessments were not conducted in a blinded manner, which may have introduced measurement bias. Ethical approval was obtained from the institutional review board (IRB #2022-4), and written informed consent was received from all participants.

**Figure 1:**
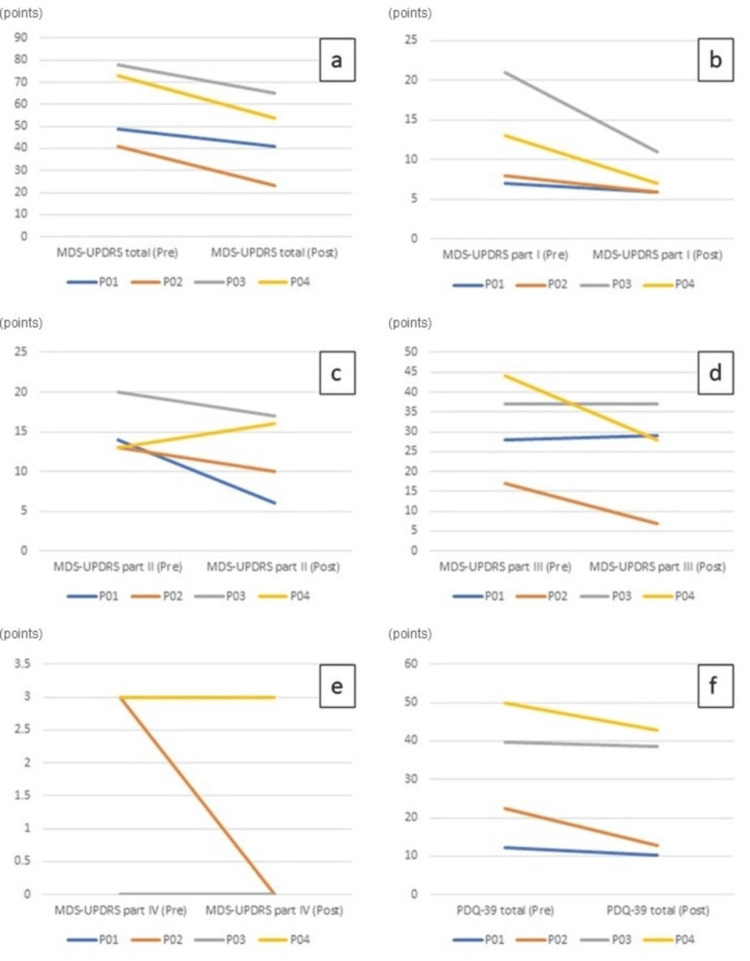
Change in MDS-UPDRS total (Ia), part I (Ib), part II (Ic), part III (Id), part IV (Ie) scores, and PDQ39 total score (If) from baseline to the end of the study. Blue, orange, gray, and yellow lines indicate Patient 1, 2, 3, and 4, respectively. MCID thresholds used in this study were as follows: MDS-UPDRS part I, 1.8 points; part II, 3.05 points; part III, 3.25 points; part IV, 0.9 points; and PDQ-39, 4.7 points, based on published anchor-based or distribution-based definitions. Individual change scores should be interpreted relative to these thresholds.

Results of the outcome measures before and after all sessions are shown in Table 2 and Figure 1. All participants were receiving physical therapy, occupational therapy, and speech therapy as part of standard care at baseline, and the physical therapy was discontinued; occupational and speech therapies were continued without modification throughout the study period. All participants completed 16 sessions as scheduled. Improvements on MDS-UPDRS part I, part II, and part III scores based on MCID were seen in 2 (P03, P04), 1 (P01), and 2 (P02, P04) participants, respectively. Two participants (P02, P04) showed improvement on the PDQ-39 summary index based on MCID, and both patients (P02, P04) also showed improvements in the PDQ-39 subitems of mobility, emotional well-being, communication, and bodily discomfort. Regarding post-event SUS, only one of the participants scored ≥70, which is generally considered acceptable by users for the system/product. SUS scores varied widely among participants (42.5, 52.5, 60, and 70), indicating heterogeneous perceptions of system usability. No participants reported adverse events such as falls, pain, or VR sickness.

**Table 2:** Outcome measures for all 4 participants before and after the 8 exercise sessions Movement Disorders Society Unified Parkinson's Disease Rating Scale = MDS-UPDRS; Parkinson’s disease questionnaire-39 = PDQ-39; System Usability Scale = SUS. Units and score directions are indicated where applicable. For PDQ-39 subscales, only changes exceeding established MCID thresholds should be considered potentially clinically meaningful; numerical fluctuations below MCID may reflect measurement variability rather than true clinical change. MCID thresholds used in this study were as follows: MDS-UPDRS part I, 1.8 points; part II, 3.05 points; part III, 3.25 points; part IV, 0.9 points; and PDQ-39, 4.7 points, based on published anchor-based or distribution-based definitions. Individual change scores should be interpreted relative to these thresholds.

		P01	P02	P03	P04
MDS-UPDRS total score	Pre	49	41	78	73
	Post	41	23	65	54
MDS-UPDRS part I	Pre	7	8	21	13
	Post	6	6	11	7
MDS-UPDRS part II	Pre	14	13	20	13
	Post	6	10	17	16
MDS-UPDRS part III	Pre	28	17	37	44
	Post	29	7	37	28
MDS-UPDRS part IV	Pre	0	3	0	3
	Post	0	0	0	3
PDQ-39 total score	Pre	12.2	22.4	39.7	50.0
	Post	10.3	12.8	38.5	42.9
Mobility	Pre	12.5	22.5	27.5	90.0
	Post	12.5	12.5	20.0	82.5
Activities of daily living	Pre	8.3	16.7	58.3	62.5
	Post	0.0	16.7	54.2	75.0
Emotional well-being	Pre	8.3	25.0	37.5	25.0
	Post	4.2	16.7	45.8	0.0
Stigma	Pre	0.0	31.3	31.3	0.0
	Post	6.3	6.3	37.5	0.0
Social support	Pre	16.7	0.0	0.0	0.0
	Post	0.0	0.0	0.0	16.7
Cognition	Pre	25.0	18.8	68.8	68.8
	Post	37.5	12.5	56.3	68.8
Communication	Pre	0.0	33.3	50.0	41.7
	Post	0.0	16.7	25.0	16.7
Body discomfort	Pre	33.3	33.3	50.0	41.7
	Post	25.0	16.7	83.3	8.3
SUS	Post only	60.0	42.5	70.0	52.5

## Discussion

This case series suggests that a VR-based table tennis program may be safe and feasible for some individuals with Parkinson’s disease, with potential clinical benefits observed in selected participants. All participants successfully completed the scheduled sessions without adverse events, indicating that this modality can be integrated into routine rehabilitation with minimal safety concerns. All VR sessions were conducted under the direct supervision of a therapist. Participants performed the VR task in a controlled environment with adequate space, and fall-risk precautions were implemented, including therapist standby and immediate assistance when needed. Participants were monitored for symptoms such as dizziness, nausea, or discomfort during and after VR sessions through clinical observation and patient self-report; however, no formal standardized cybersickness questionnaire was administered. The absence of formal screening tools for cybersickness or dizziness represents a limitation and should be addressed in future studies using standardized safety assessments.

Compared with previous VR-based rehabilitation studies in PwPD, which have primarily employed balance training, gait training, or task-oriented activities [10,11], our program introduces a novel form of sport-simulation-based VR training. Table tennis inherently requires rapid visuomotor integration, anticipatory postural adjustments, and upper-limb coordination, all of which are domains frequently impaired in PwPD. Thus, VR table tennis may leverage disease-relevant motor circuits more directly than conventional VR exercises.

Given the completion rate and absence of adverse events, our VR table tennis program demonstrated its efficacy as a safe and feasible rehabilitation method for PwPD. During interviews after the study, the participants noted that this program encouraged participation, which could potentially improve continuity of rehabilitation for PwPD. In addition, VR rehabilitation can be a useful method in environments where isolation is necessary due to specific reasons, such as the spread of infections, although this program was not conducted in isolated circumstances [12]. Our VR table tennis program may also be able to improve mood disorders in addition to improving motor function, which would then have a positive effect on the ability to continue with an exercise in PwPD. This suggests positive effects in both the short term and long term for PwPD.

An important finding of this study is that clinically meaningful improvements were observed in motor and QOL measures for some participants, as determined using MCID thresholds. Although the small sample size precludes firm conclusions regarding efficacy, these preliminary results are consistent with prior reports demonstrating that VR-based rehabilitation can enhance motor function, balance, emotional well-being, and activities of daily living in PwPD [10,11]. The observed benefits may be mediated through several mechanisms: (1) increased exercise intensity facilitated by immersive environments; (2) enhanced motivation and engagement due to gamification elements; and (3) repeated sensorimotor practice fostering neuroplastic changes. Sport-simulation VR, such as table tennis, may further enhance anticipatory movement strategies and visuomotor processing, aspects that are less emphasized in standard rehabilitation protocols. However, it is noted that changes in motor and QOL measures varied across participants, indicating heterogeneous individual responses. Taken together, the primary outcome did not demonstrate a consistent signal across participants. The observed improvements were driven by a subset of individuals rather than reflecting a uniform response to the intervention.

In addition to motor function, some participants showed improvements in emotional well-being and communication domains of the PDQ-39. VR interventions have been proposed to promote psychological benefits by providing enjoyable and self-directed activities, reducing exercise-related anxiety, and offering a sense of achievement [4]. These effects may contribute to improved adherence, a critical factor in maintaining long-term physical activity in PwPD, who often face barriers such as apathy, depression, or motor fluctuations.

SUS scores varied widely among participants (42.5, 52.5, 60, and 70), indicating heterogeneous perceptions of system usability. Interestingly, the participant who achieved a SUS score above 70 did not have the highest cognitive score or the mildest disease stage. This finding suggests that VR usability may not be solely determined by age, disease severity, or global cognitive function, but may also reflect individual adaptability and prior experience coping with motor limitations.

The limitations of this pilot study include the small number of participants and its being a single-group study. Other limitations include that the safety of this program in a home setting has yet to be determined, and that the effects of our program could also be influenced by occupational and speech therapies. MDS-UPDRS assessments were conducted by clinicians involved in routine patient care, and blinding was not feasible. Therefore, the possibility of measurement bias due to non-blinded scoring cannot be excluded and should be considered when interpreting the results. The observed improvements should be interpreted as the effect of substituting conventional physical therapy with VR-based rehabilitation in the context of ongoing occupational and speech therapy, rather than as an isolated effect of VR table tennis alone. Because occupational and speech therapies were maintained throughout the study period, their contributory effects cannot be entirely excluded; however, their stable implementation before and during the intervention period makes it less likely that the observed changes were driven by these therapies alone. Another limitation was that MCID values were used to indicate clinical meaningfulness of observed changes and were not intended as measures of validity or confirmatory endpoints. We also acknowledge the following limitation: the absence of objective monitoring of exercise intensity and energy expenditure limited the precise characterization of the training dose. The last limitation is that all participants needed time to become familiar with the VR equipment.

## Conclusions

VR-based table tennis appears to be a safe and feasible rehabilitation exercise for selected individuals with Parkinson’s disease. Given the importance of ongoing physical activity in this population, this approach may represent a potential option to support engagement in exercise across different settings; however, its effects on motor function and quality of life require confirmation in larger, controlled studies.
